# Renal injuries in conflict zones: a 6-year study of traumatic cases in Afghanistan

**DOI:** 10.1186/s13031-023-00566-1

**Published:** 2024-01-06

**Authors:** Tomasz Ząbkowski, Robert Brzozowski, Adam Daniel Durma

**Affiliations:** 1 Department of Urology, Miliary Institute of Medicine – National Research Institute, Warsaw, Poland; 2 Department of General and Oncological Surgery, 5th Military Clinical Hospital with Polyclinic, Cracov, Poland; 3 Department of Endocrinology and Radioisotope Therapy, Miliary Institute of Medicine – National Research Institute, Warsaw, Poland

**Keywords:** Traumatic renal injuries, Combat trauma, Penetrating trauma, Treatment strategy

## Abstract

**Purpose:**

During hostilities, gunshot wounds are the most common cause of penetrating injuries. In 8–10% of abdominal injuries kidneys are involved. The treatment method include surgical or conservative treatment (fluids + blood components).

**Methods:**

Of 1266 combat trauma cases treated during 6 to 14 rotation of the Polish Military Contingent in Afghanistan, we extracted a subgroup of 44 kidney injuries. Corelation of trauma mechanism, PATI score, treatment methods, and outcomes was evaluated.

**Results:**

Out of the 41 renal injuries, 20 considered left, 18 right, and 3 both kidneys. There were no statistical significancy in injury lateralization (*p* = 0.669), and no differences regarding side of a trauma and quantity of blood component used for the treatment (*p* = 0.246). Nephrectomy was performed on 17 patients (13 left vs. 4 right). A significant correlation between PATI score and the need for a nephrectomy (*p* = 0.027) was confirmed. Penetrating trauma recquired higher number of blood components comparing to blunt trauma (*p* < 0.001). The renal salvage rate was in study group was 61.36%. The overall survival (OS) rate was 90.25% − 4 patients died due to trauma.

**Conclusions:**

The damage side does not result in a statistically significant increase in the need for blood transfusions or differences in the PATI score. The mechanism of trauma does, however, affect the number of blood components required for treatment, particularly in cases of penetrating trauma. With the introduction of proper treatment, the overall survival rate exceeds 90%, even when opting for conservative treatment.

## Introduction

Military service is one of the most dangerous occupation in the world. During war or hostilities, the risk of trauma (or even death) is higher than in any other jobs. Renal trauma occurs in 1–5% of all trauma cases [1–3]. Some studies report even higher numbers of renal injuries in abdominal traumas [4]. The kidney is regarded as the genitourinary organ that experiences the highest frequency of injuries, with a ratio of 3 males to 1 female [5]. In times of conflict, gunshot wounds are the primary causes of penetrating injuries and tend to be more severe and less predictable compared to blunt trauma. This is primarily due to the significant amount of kinetic energy transferred to the tissues and the unpredictable path the projectile takes within the human body [6–9]. Abdominal gunshot wounds typically result in parenchymal destruction and are frequently accompanied by injuries to multiple organs [10, 11]. Penetrating or even blunt injury not only contributes to direct damage of the parenchymal organs, but also can cause injury in the mechanism of vascular damage and blood loss [12]. Minor low-velocity gunshot wounds can often yield favorable outcomes with conservative treatment [13–15]. In cases of high-velocity gunshot injuries that result in significant and extensive tissue damage, a nephrectomy may be necessary, despite efforts to preserve the kidney through conservative treatment [16]. According to several studies, non-surgical treatment has shown success rates of approximately 50% for stab wounds and up to 40% for gunshot wounds in stable patients [17]. On average, around 8–10% of abdominal injuries, both blunt and penetrating, involve the kidneys. Penetrating renal trauma is often accompanied by associated injuries, with incidence rates ranging from 77 to 100%. Gunshot wounds tend to result in a higher frequency of injury to adjacent organs compared to stab wounds. Most patients with penetrating renal trauma also sustain injuries to adjacent organs, further complicating treatment. The small intestine is the organ most commonly affected, although the specific percentage of injuries varies across studies [6, 18, 19]. The available data indicates that the presence of multiorgan injuries, in the absence of an expanding hematoma and hemodynamic instability, does not elevate the risk of requiring a nephrectomy [20]. With the global increase in wars, conflicts, extremism, and acts of terrorism, blast or bullet abdominal injuries are becoming more common and present a significant surgical challenge. It is important to note that such injuries not only affect military personnel but also civilians as a result of actions in war zones [21]. The severity and extent of gunshot damage are determined by the force of impact of the bullet or projectile, which is mainly dependent on its velocity. Gunshot injuries are categorized as high-energy or low-energy based on the extent of tissue damage they inflict [22]. Penetrating trauma is further classified according to the velocity of the projectile, with high-velocity (e.g., rifle bullets: 800–1000 m/sec), medium-velocity (e.g., handgun bullets: 200–300 m/sec), and low-velocity items (e.g., knife stabs) [24]. The kinetic energy of a bullet, calculated as half its mass multiplied by the square of its velocity (KE = mv^2^/2), is significantly higher for high-velocity bullets compared to low-velocity ones. Consequently, rifle bullets possess a greater potential for causing wounds than handgun bullets [25]. However, the specific projectile type, tissue affected, and distance between the weapon and the victim also play crucial roles in determining the nature of these injuries [9]. The size of a renal injury can be represented as U(t) = KME(t)/Os, where U(t) signifies the size of the injury, “K” denotes the caliber, “M” represents the load power in the bullet, “E(t)” symbolizes the bullet’s energy, and “Os” indicates the distance of the gunshot. Higher projectile caliber, its load power, bullet speed are reflecting the energy of the injury and corresponds to a larger injury size.

The primary objective of this study was to analyze traumatic renal injuries and determine the distribution of the most common abdominal injuries, aiming to answer the question: “Does the side of injury affect its extent and the subsequent increased need for blood products?“. The secondary question was “Does the soldier combat posture can influence the extent of the injury”.

## Materials and methods

The study conducted from September 2009 to March 2014 involved a total of 1266 combat trauma patients treated in two health centers located in the Forward Operating Base, Ghazni, Afghanistan. The patients were not personally involved or identifiable. This study specifically focused on patients treated during the 6 to 14 rotation of the Polish Military Contingent, including the Medical Support Team Role 2 and Forward Surgical Team. Among the patients, 41 individuals (3.23% of the total) had combat-related renal injuries and were included in the study. A retrospective review of their medical history and treatment outcomes was conducted. The study group consisted of 40 male soldiers (97%) and 1 female soldier (3%). The median age of the patients was 29 years. In the majority of cases, renal injuries occurred alongside injuries to multiple organs. Table 1 presents basic epidemiological data of the group.

**Table 1:** Data of the patients with renal injuries

	Data	n = 41
Gender	Female	1 (3%)
	Male	40 (97%)
Age	Median	29
	Mean	28.61
	Range	19–50
	SD	7.34

SD- standard deviation

**Fig. 1:**
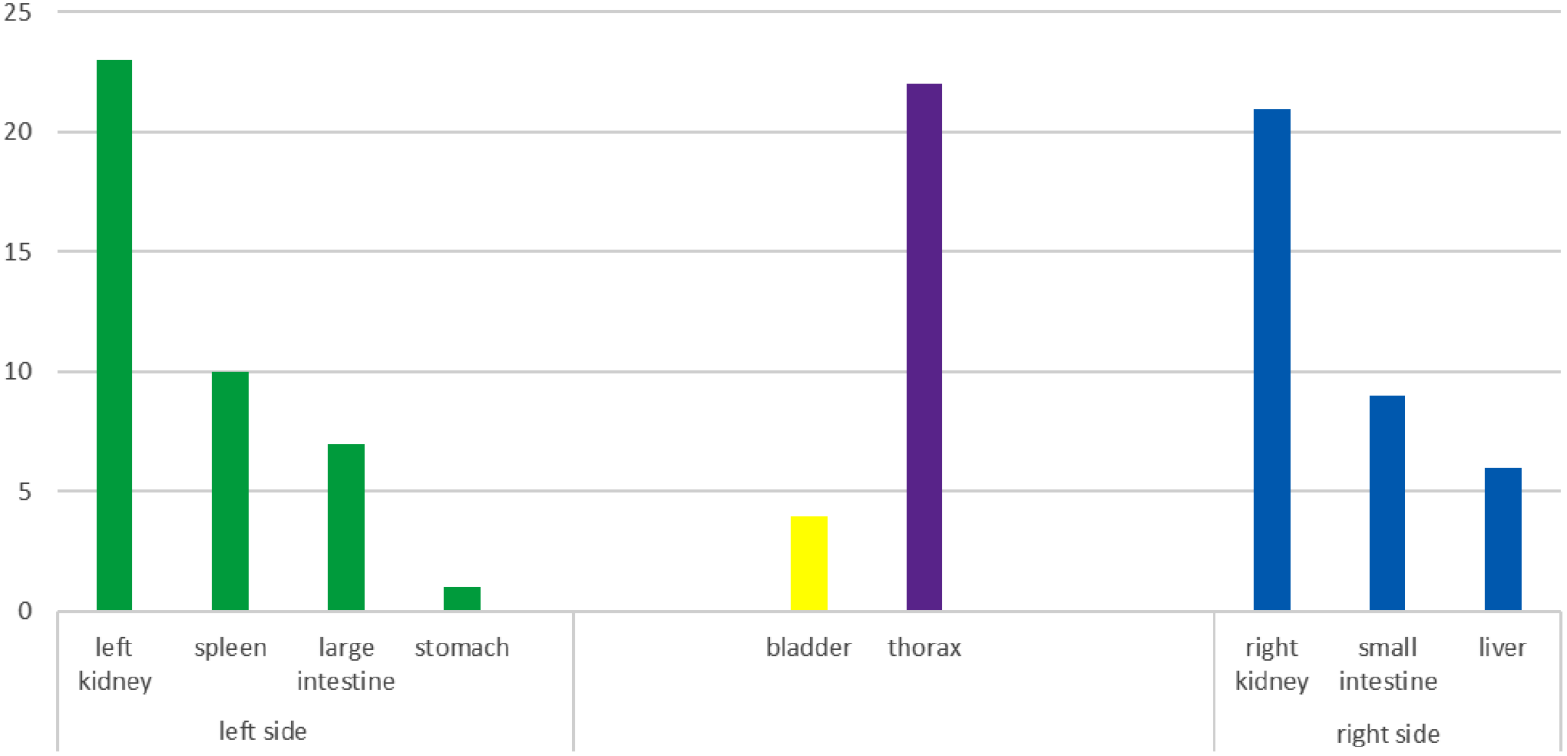
Multiorgan injuries with regard to left and right side of abdomen

We evaluated the following aspects of kidney injury: the cause or mechanism of trauma, the Penetrating Abdominal Trauma Index (PATI) score in cases of penetrating trauma, the treatment approach, and the presence of multiorgan injury. PATI scores were calculated during the perioperative period to determine the appropriate treatment method and to predict potential postoperative complications.

In a battlefield setting where access to computed tomography is limited, a chest X-ray and a focused assessment with sonography for trauma (FAST) exam were performed [26]. The aim was to rapidly assess potential abdominal cavity damage by detecting the presence or absence of blood in the pericardium or peritoneum in unstable patients. It is important to note that a negative ultrasound result does not completely rule out the possibility of intra-abdominal organ injuries, especially injuries to hollow organs. Therefore, the ultrasound examination was always interpreted in conjunction with the clinical condition of the patients and repeated if necessary.

The “Apache shelling” presented in Table 2 as one of the injury mechanism refers to bullet fragment/ricochet from AH-64 Apache helicopter fire, while “HESCO” refers to fall from HESCO Concertainer which is a military gabion made of a collapsible wire mesh container combined with heavy duty fabric liner – one of basic structures used for fortification of outpost and military bases.

**Table 2:** Detailed data of the patients

Patient No.	Age	Gender	Battle (BT) / Non Battle (NBT)	Mechanism	Kidney R/L	Blunt (B)/ Penetrating (P)	PATI	AAST	Type of trauma	Treatment	Blood transfusions
1	42	M	BT	GSW	L	P	54	V	HF	LN, Conservative	4U pRBC, 4U FFP, 6U FWB, 10U Cryo
2	19	M	NBT	MVA	R	B	0	III	HE	conservative	NO
3	26	M	NBT	HESCO	R	B	0	II	LE	conservative	NO
4	20	M	BT	IDF	R	P	15	IV	LV	NSS	3U pRBC, 3U FFP, 6U FWB, 10U Cryo
5	22	M	BT	GSW	R	P	22	III	HV	NSS	4U pRBC, 4U FFP, 6U FWB, 10U Cryo
6	21	M	BT	GSW	L	P	34	V	HV	LN	4U pRBC, 4U FFP, 8U FWB, 10U Cryo
7	21	M	BT	GSW	R	P	9	II	HV	RL-drainage	3U pRBC, 3U FFP, 10U Cryo
8	23	M	BT	mine blast	R	P	19	II	LV	RL-drainage	3U pRBC, 3U FFP, 5U FWB, 10U Cryo
9	27	M	BT	IED	L	P	54	IV	LV	LN	5U pRBC, 5U FFP, 10U FWB, 10U Cryo
10	40	M	BT	Apache shelling	R	P	36	III	LV	NSS	3U pRBC, 3U FFP, 4U FWB, 10U Cryo
11	24	M	BT	mine blast	L	P	44	V	LV + HE	LN	4U pRBC, 4U FFP, 8U FWB, 10U Cryo
12	50	M	BT	shooting	L	P	20	V	HV	LN	4U pRBC, 4U FFP, 4U FWB, 10U Cryo
13	22	M	BT	Apache shelling	L	P	54	IV	HV	LN	5U pRBC, 5U FFP, 10U FWB, 10U Cryo
14	23	M	BT	RPG	R	P	11	IV	LV	RN	NO
15	23	M	BT	IED	R	P	25	IV	LV	suturation of right renal vein with venous patch	3U pRBC, 3U FFP, 8U FWB, 10U Cryo
16	43	M	BT	IED	R	P	19	II	LV	RL-shrapnel remove from kidney	3U pRBC, 3U FFP, 5U FWB, 10U Cryo
17	21	M	NBT	MVA	R	B	0	II	LE	conservative	NO
18	27	M	BT	GSW	L/R	P	63	IV/II	HV	LN; suturation of right kidney, drainage	4U pRBC, 1U WBC, 3U FFP, 4U Cryo
19	42	M	BT	shooting	L	P	24	IV	HV	LN	3U pRBC, 3U FFP, 5U Cryo
20	27	M	BT	IED	R	P	19	II	LE	conservative	4U pRBC, 4U FFP, 8U FWB, 10U Cryo
21	34	M	NBT	MVA	L	B	0	II	LE	conservative	NO
22	32	M	BT	IED	R	P	28	III	HE	RN	5U pRBC, 5U FFP, 10U Cryo
23	27	M	BT	IED	L/R	B		III	HE	conservative (right kidney); suturation of left renal parenc	6U pRBC, 5U FFP, 4U FWB, 10U Cryo
24	24	M	BT	GSW/mine blast	L	P	37	IV	HE	LN	3U pRBC, 3U FFP, 4U FWB, 10U Cryo
25	23	M	BT	IED	L	P	58	V	HE	LN, packing	5U pRBC, 5U FFP, 4U FWB
26	34	M	BT	IED	L	P	61	IV	HE	suturation and drainage	4U pRBC, 4U FFP, 5U FWB, 10U Cryo
27	25	M	BT	IED	R	P	49	IV	HE	RN	4U pRBC, 4U FFP, 4U FWB, 10U Cryo
28	29	M	BT	IED	L	P	51	IV	HE	LN	4U pRBC, 4U FFP, 4U FWB, 10U Cryo
29	26	M	BT	IED/mine blast	L	B	0	III	HE	suturation of renal left parenchyma	3U pRBC,3U FFP
30	23	M	BT	IED/mine blast	L/R	B	0	II/III	LE	conservative	NO
31	32	M	BT	IED	L	P	33	II	HE	conservative	4U pRBC, 4U FFP, 4U FWB, 10U Cryo
32	29	F	NBT	MVA	L	B	0	II	HE	conservative	NO
33	28	M	BT	RPG/mine blast	L	P	0	II	HE	conservative	NO
34	28	M	BT	RPG	R	P	14	II	HE	RL-shrapnel remove and suturation	3U pRBC, 3U FFP
35	31	M	BT	IED	L	P	36	III	HE	LN	4U pRBC, 4U FFP, 4U FWB, 10U Cryo
36	24	M	NBT	MVA	L	B	0	II	HE	conservative	NO
37	25	M	BT	rocket shelling	L	P	13	III	LE	suturation, drainage	3U pRBC, 3U FFP
38	27	M	NBT	MVA	R	B	0	II	HE	conservative	NO
39	38	M	BT	RPG	R	P	25	III	HE	suturation, packing	4U pRBC, 4U FFP, 4U FWB, 10U Cryo
40	29	M	BT	IED	L	P	55	III	HE	LN	5U pRBC, 5U FFP, 4U FWB, 10 U Cryo
41	42	M	BT	GSW	R	P	25	IV	HE	RN	4U pRBC, 4U FFP, 4U FWB, 10U Cryo

M- male, F- female, BT = battle trauma; NBT = non-battle trauma; L = left; R = right; B = blunt; *P* = penetrating; LN = left nephrectomy; RN = right nephrectomy; RL = right lumbotomy; HV = high-velocity; LV = low-velocity; HE = high-energy; LE = low-energy; GSW- gunshot wound; IED- improvised explosive device, RPG- rocket propelled grenade; MVA- motor vehicle accident, U- units, pRBC- packet red blood cells, FFP-fresh frozen plasma, FWB- fresh whole blood, Cryo- cryoprecipitate, WBC- white blood cells; PATI- penetrating abdominal trauma index; AAST – American Association for the Surgery or Trauma scale, HESCO- fall from HESCO Bastion

**Fig. 2:**
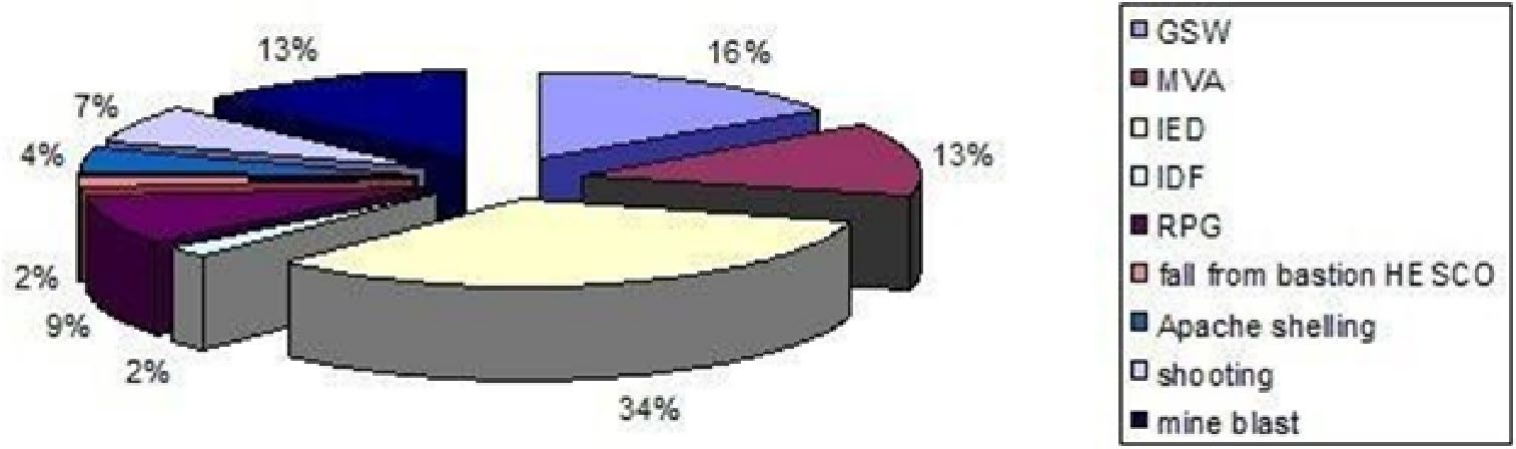
Mechanism of injury

**Fig. 3:**
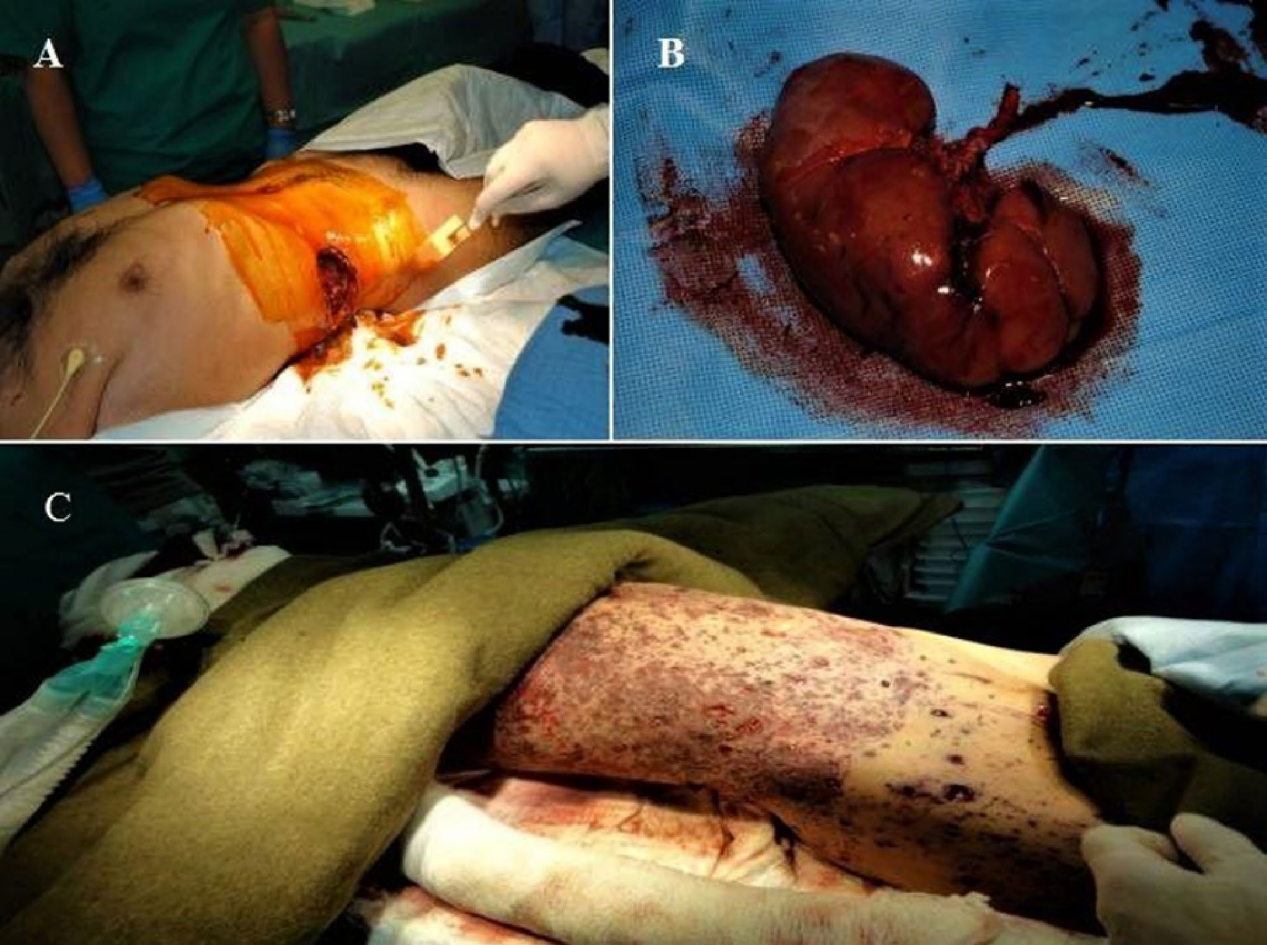
**A** Shrapnel wound of abdomen with kidney rupture of the right side. **B** Gunshot wound with the left renal vascular pedicle injury. **C** Loculated shrapnel injuries after IED

### Statistical analysis

Statistical analyses were performed using Stata/IC 14.2 software. The Kaplan-Meier survival analysis was utilized to assess the renal salvage rate, with the PATI score serving as an indicator for the need for nephrectomy. The results were visualized on a graph, displaying hazard ratios and 95% confidence intervals.The corelation between trauma side and PATI score/number of blood component used was performed with use of Shapiro-Wilk test to verify whether the results met the rules of normal distribution, followed by analyzed using appropriate tests, such as the t-Student test, Wilcoxon test, and Mann-Whitney test. A significance level of *p* < 0.05 was adopted.

**Fig. 4:**
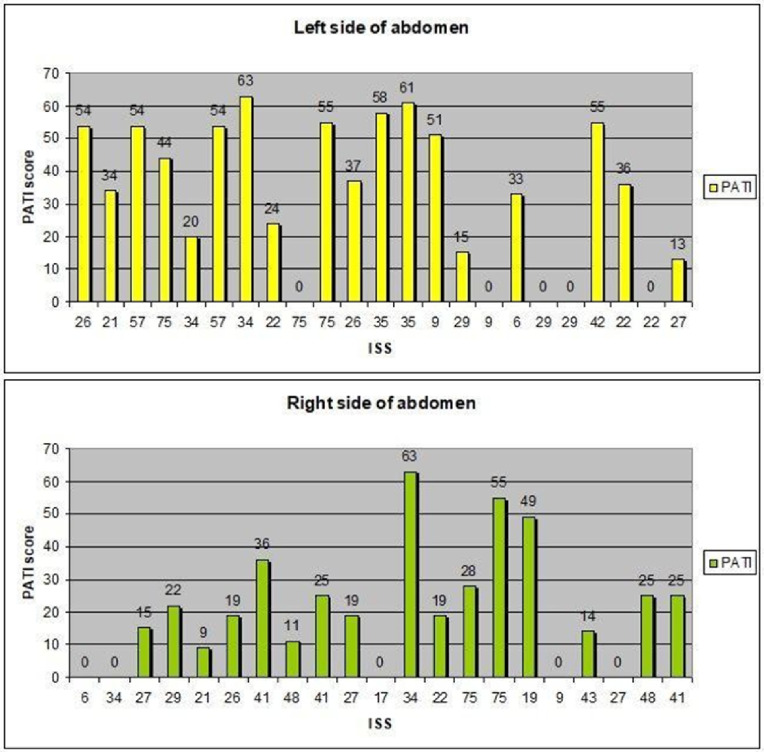
Correlation between PATI score and AAST scale

**Fig. 5:**
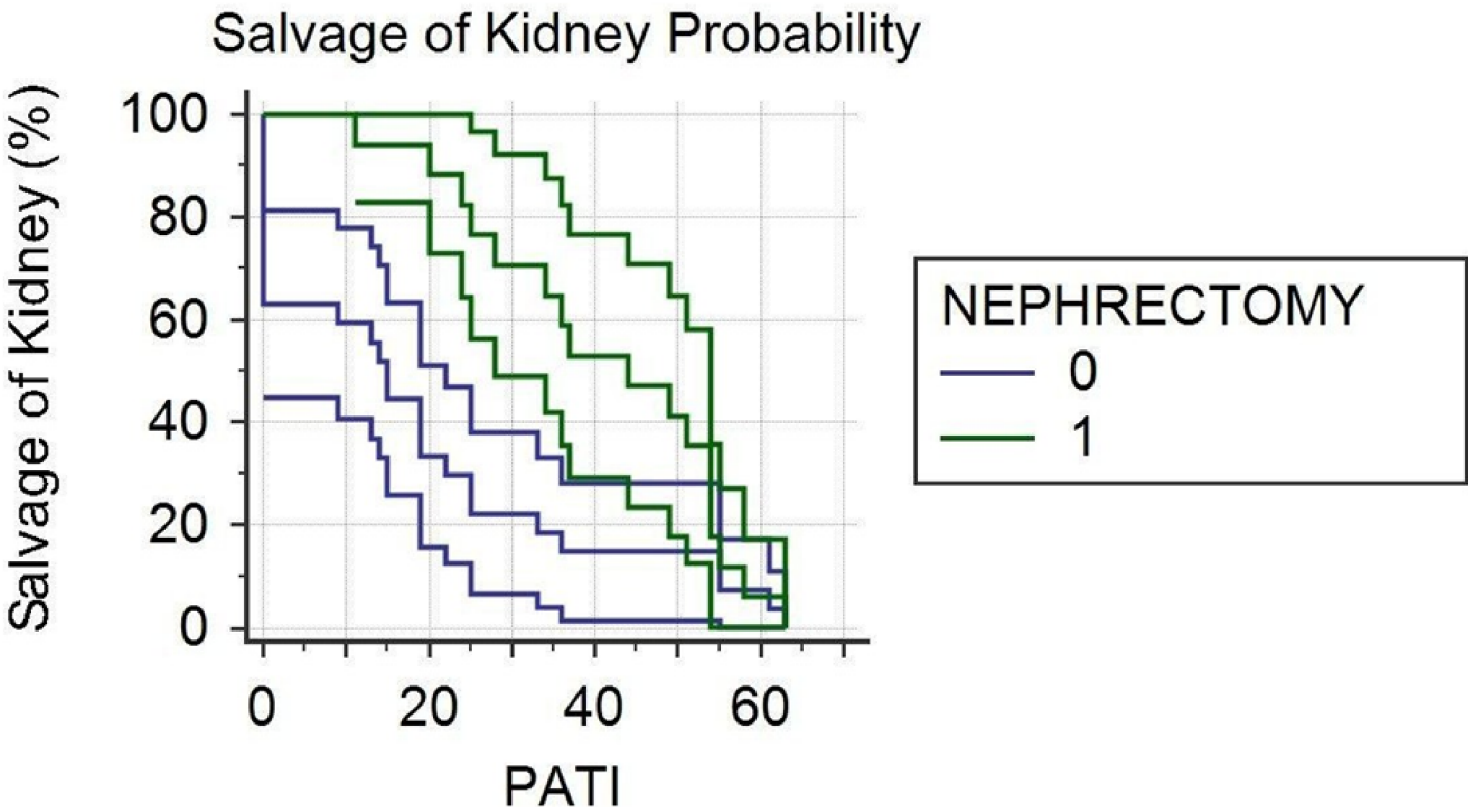
Salvage of kidney probability

## Results

Out of the 41 patients in the group, a total of 44 renal injuries were confirmed. Among these injuries, 21 were observed in the right kidney, 23 were observed in the left kidney while 3 patients had an injury of both kidneys. In most cases, renal trauma occurred alongside multiorgan injuries. Additional organs affected by the injuries included the spleen (n = 10), small bowel (n = 9), large bowel (n = 7), bladder (n = 4), liver (n = 6), thorax (n = 22), lower extremities (n = 14), upper extremities (n = 7), and head (n = 8) (Fig. 1).

Regarding the trauma cases, 37 instances (84.09%) were attributed to battle injuries, while 7 cases (15.90%) were classified as non-battle injuries. Among the renal trauma cases, 32 were caused by penetrating trauma, while 12 were a result of blunt trauma. The mechanisms leading to renal trauma included Gunshot Wound (GSW) in 7 patients (15.90%), Improvised Explosive Device (IED) in 15 patients (34.09%), Motor Vehicle Accident (MVA) in 6 patients (13.63%), Apache shelling in 2 patients (4.54%), mine blast in 6 patients (13.63%), Indirect Fire (IDF) in 1 patient (2.27%), Rocket Propelled Grenade (RPG) in 4 patients (9.09%), fall from bastion HESCO in 1 patient (2.27%), and shooting in 3 patients (6.81%) (refer to Figs. 2 and 3).

In addition, injuries were classified as being of high-velocity (9 patients), low-velocity (8 patients), low energy (6 patients) and high-energy (21 patients). A PATI score of 9–63 was observed in patients with grade II injury, 22–55 with grade III injury, 15–63 with grade IV injury, and 20–58 with grade V injury (Fig. 4).

Ten patients had Grade II renal injuries and two patients had Grade III renal injuries. Conservative treatment was administered for all cases. Two patients with Grade III injuries and one patient with Grade IV injury underwent Nephron Sparing Surgery (NSS). Among the three patients with Grade II injuries, surgical revision was performed in the right retroperitoneal space, treating the perirenal hematoma, without any visible renal injuries, and drainage was conducted. The patient with Grade IV injury received suturing of the right renal vein with a venous patch. Saturation of the renal parenchyma was carried out in four patients with Grade III injuries and one patient with Grade IV injury, along with excision of shrapnel from the renal parenchyma. Nephrectomy was performed in 17 patients, including 13 left kidney nephrectomies (6 with Grade IV injuries and 5 with Grade V injuries), and four right kidney nephrectomies (3 with Grade IV injuries and 1 with Grade III injury). In total, 31 out of 41 patients required blood transfusions as follows: 3–6 units of packed Red Blood Cells (pRBC), 3–5 units of Fresh Frozen Plasma (FFP), 4–10 units of Fresh Whole Blood (FWB), and 10 units of Cryoprecipitate (Cryo). The renal salvage rate was 61.36%, and the overall survival rate was 90.24%. There were four casualties (9.75%) among patients classified as Battle injury died of wounds (DOW). Statistical analysis, including a Kaplan-Meier survival curve, revealed a significant correlation between the PATI score and the necessity for nephrectomy (*p* = 0.0269), as well as a relatively high rate of kidney salvage using conservative treatment (Fig. 5).

The combined and detailed data of all patients analyzed in the study are presented in Table 2 below.

Analysis showd that there is no statistical differences in PATI score and quantity of blood components (BC) used for patients treatment (*p* = 0.170 and *p* = 0.246 respectively) (Table 3). Neverheless we have noticed a significat increase of BC quantity used for treatment of patients with penetrating trauma compared to ones with blunt injuries (*p* < 0.001) (Table 4).

**Table 3:** Comparison of the amount of blood components used for treatment and the PATI score depending on the side of the injury

	L (n = 20)		R (n = 18)		*p*
	M	SD	M	SD	
Blood components quantity	16.40	10.44	14.78	10.02	0.246
PATI	31.40	22.06	18.05	12.91	0.170

**Table 4:** Comparison of the amount of blood components used depending on the trauma type

	P (n = 31)		B (n = 10)		*p*
	M	SD	M	SD	
Blood components quantity	19.35	7.6	3.10	7.52	< 0.001

## Discussion

In our study we did not show that there is a statistical difference in occurrence of one-sided damage. Out of the 41 renal injuries, 20 were on the left kidney, 18 were on the right one, and 3 was on both sides, thus the soldier combat posture possibly do not influence the location of the injury. There were also no statistical differences regarding side of a trauma and quantity of blood component used for the treatment, however left-sided injuries required 16.4 ± 10.44 units of blood components, while right sided required 14.78 ± 10.02 units.

Nephrectomy was performed on 17 patients: 13 had left kidney nephrectomy and 4 had right kidney nephrectomy, which can explain the mentioned reasons of the differences in blood components quantity used durging treatment. We additionaly have noticed a significant correlation between higher PATI score and the higher need for a nephrectomy procedure.

In study group the renal salvage rate was in study group was 61.36%, and the overall survival rate was 90.25%. Only four of our patients (9.75%) died due to battle injury wounds, mostly due of politrauma. The spleen was additionally the most common injured abdominal organ, (n = 10), followed by the small intestine (n = 9). Obtained results points that the treatment of kidney (battle or non-battle) injury gives relatively high percentage of successful procedures, even in extreme conditions of war and battlefield. The most important result of our observation is that penetrating trauma recquire higher number of blood components comparing to blunt trauma (*p* < 0.001). Although this is not a suprising fact, and potentiall unpredictable route of projectiles in human body can give higher number of organs damaged, the information about trauma cause can help to prepare medical staff for proper and idividualized treatment of the casuality. The extent of tissue damage caused by penetrating bullets depends on the intensity of impact, leading to a diverse range of ballistic injury patterns resulting from complex interactions between the projectile and various tissues. Understanding the specific mechanisms that contribute to increased tissue destruction can aid in identifying less frequent injuries caused by high-intensity impacts, which are also associated with a higher risk of infectious complications [27]. Taş et al. found that injuries to the left kidney occurred more frequently than injuries to the right kidney. However, the mean PATI score, the number of injured organs, and the requirement for blood transfusions did not differ based on the side of the injury. Various scientific publications present differing opinions regarding the side of renal injury. In cases of blast effects, it was observed intraoperatively that the liver provides protective effects to the right kidney, enabling it to withstand the impact of the blast injury. In instances of renal and liver injuries not directly caused by a projectile, it was noted that higher-grade liver injuries occurred due to the blast effect, while lower-grade injuries to the right kidney happened concurrently [28]. Voelzke et al. presented a study involving 201 patients (206 renal units) with renal gunshot wounds, of which 96.5% (194 out of 201) had multi-organ injuries, with over 74.6% involving more than one organ. The liver was the most commonly injured organ. Ninety-five renal units (excluding nephrectomy) with associated small or large bowel injuries underwent repair. The renal salvage rate was 85.4%, and out of the 206 renal units, 30 required nephrectomy. The overall survival rate (OSR) was 90.6%, with two deaths occurring during the operation and 17 deaths in the postoperative period [29]. High OSR was also confirmed in other studies [14, 30]. However, the experience of some centers shows that the majority of gunshot abdominal wounds with renal injury can be safely managed non-operatively [31–33]. It is crucial to note that these results were obtained in non-hostile, peaceful conditions, and a direct comparison of these results to those obtained in our study may not be accurate. Complex genitourinary injuries often occur in conjunction with lower extremity injuries, amputations, as well as pelvic and abdominal wounds in military combat operations. The management strategies employed greatly influence the nature of combat-related genitourinary injuries. For instance, urinary system or genitalia wounds account for approximately 5% of all combat injuries. In cases primarily involving penetrating injuries, immediate attention is necessary to preserve viable tissue [34]. Paquette conducted research on 2712 trauma cases, of which 76 (2.8%) included one or more genitourinary injuries. Among the 29 kidney injuries, 2 were explored but did not receive further treatment, 7 were placed under observation, 1 was repaired, and 19 casualties necessitated nephrectomy [35]. Medical personnel should also be mindful of the indirect outcomes of trauma that can lead to renal injury. Damage to musculoskeletal tissue resulting in rhabdomyolysis can cause acute kidney injury (AKI) [36]. Vascular injury followed by blood loss and reduction in circulating blood volume can also be a cause of AKI [37]. It is important to note that abdominal gunshot wounds with no exit wound can result in thoracic injuries as well [38]. The function of kidneys after trauma in military casualties was evaluated by World [39]. Records of 287 patients with severe injury were examined to identify predictive factors of renal dysfunction. Results showed that the best predictors of renal function were pulse rate and body temperature, but the prediction was overly optimistic at lower estimated glomerular filtration rate (eGFR) values. Present studies show that new biomarkers of kidney injury, such as kidney injury molecule (KIM-1) or tissue inhibitor metalloproteinase 2 and insulin-like growth factor binding protein 7 (TIMP2 × IGFBP7), have the potential to be useful in the assessment of AKI, although further research is necessary to assess their usefulness in gunshot trauma patients [40, 41].

## Conclusions

In the battlefield, gunshot wounds are the most common cause of penetrating injuries and are more severe and less predictable than blunt trauma. While the left side of the abdomen is slightly more susceptible to trauma, the specific side of the damage does not lead to a statistically significant increase in the need for blood transfusions or differences in the PATI score, as well as treatment predictions. However, the mechanism of trauma, particularly in cases of penetrating injuries, does affect the number of blood components required for treatment, leading to an increase. The position of the soldier may potentially influence the frequency of unilateral injuries, but the results were not statistically significant. Individualization of treatment is crucial for survival, and having an experienced surgical team results in an overall survival rate of over 90%, even when conservative treatment with proper fluid infusion is introduced.

## Data Availability

The datasets used and/or analyzed during the current study are available from the corresponding author on reasonable request.
